# Optimised extreme gradient boosting model for short term electric load demand forecasting of regional grid system

**DOI:** 10.1038/s41598-022-22024-3

**Published:** 2022-11-11

**Authors:** Zhao Qinghe, Xiang Wen, Huang Boyan, Wang Jong, Fang Junlong

**Affiliations:** 1 Electrical Engineering and Information College, Northeast Agricultural University, Harbin, China; 2 Economic and Technological Research Institute of State Grid Heilongjiang Electric Power Co., LTD, Harbin, China

**Keywords:** Energy science and technology, Engineering, Mathematics and computing

## Abstract

Load forecast provides effective and reliable guidance for power construction and grid operation. It is essential for the power utility to forecast the exact in-future coming energy demand. Advanced machine learning methods can support competently for load forecasting, and extreme gradient boosting is an algorithm with great research potential. But there is less research about the energy time series itself as only an internal variable, especially for feature engineering of time univariate. And the machine learning tuning is another issue to applicate boosting method in energy demand, which has more significant effects than improving the core of the model. We take the extreme gradient boosting algorithm as the original model and combine the Tree-structured Parzen Estimator method to design the TPE-XGBoost model for completing the high-performance single-lag power load forecasting task. We resample the power load data of the Île-de-France Region Grid provided by Réseau de Transport d’Électricité in the day, train and optimise the TPE-XGBoost model by samples from 2016 to 2018, and test and evaluate in samples of 2019. The optimal window width of the time series data is determined in this study through Discrete Fourier Transform and Pearson Correlation Coefficient Methods, and five additional date features are introduced to complete feature engineering. By 500 iterations, TPE optimisation ensures nine hyperparameters’ values of XGBoost and improves the models obviously. In the dataset of 2019, the TPE-XGBoost model we designed has an excellent performance of MAE = 166.020 and MAPE = 2.61%. Compared with the original model, the two metrics are respectively improved by 14.23 and 14.14%; compared with the other eight machine learning algorithms, the model performs with the best metrics as well.

## Introduction

Load forecasting is a technique used by the energy-providing utility to predict the electrical power needed to meet the demand and supply equilibrium^1^. The technique can provide a reference for the daily operation of regional power grids and the formulation of dispatching plans. According to the results of power load forecasting, dispatchers can reasonably coordinate the distribution of the output of each power plant, maintain a balance between supply and demand, and ensure power grid stability. This determines the start-stop arrangement of the generator set, reduces the redundant generator reserve capacity value, and reduces the power generation cost^2,3^. Time series forecasting with the Machine Learning technique is the application of a model to predict future values through experience and by the use of previously observed values automatically. In recent years, power load forecasting combining machine learning methods, as a special sequence with stable data sources from grid operators or energy utilities, has broad research prospects: Muzumdar etc. propose a mixed model for consumer’s short-term load forecasting, which contained random forest, support vector regressor, and long short-term memory as base predictors to handle varying traits of energy consumption^4^. Deng etc. proposes a Bagging-XGBoost algorithm for short-term load forecasting model, which can warn the time period and detailed value of peak load of distribution transformer^5^. Chen etc. proposes a short-term load forecasting framework integrating a boosting algorithm and combined a hybrid multistep method into the single-step forecasting^6^. Tan etc. proposes a Long Short-Term Memory (LSTM) network based hybrid ensemble learning forecasting model for short-time industrial power loading forecast^7^. Xian etc. proposed a multi-space collaboration (MSC) framework for optimal model selection to finish the model selection with strong adaptability to in more candidate size of the parameter domain^8,9^.

From a technical point of view, the regional power grid load has stronger periodic stability than smaller systems or bus-systems, but it has less periodicity than larger national-level systems^2^. While from an application point of view, for the interconnection and transaction of the national or cross-border grid, the regional power grids are the smallest node unit. So, there is practical value to research its loading forecasting research on power systems. On the other hand, from the perspective of the development of the machine learning algorithm, all core model is usage and powerful enough in pure theory, especially for the boosting ensemble algorithms. But the feature engineering of the dataset or data and hyperparameters tuning of the models themselves will affect the application results greatly. However, the features of dataset and tuning process is interdependent and there is less research now on boosting methods for timeseries without external variable, which (external variable) in some application environment have more importance and the impact of the time series itself on forecast performance would be masked^10^.

Gradient boosting is a state-of-the-art Machine learning algorithm. The Extreme Gradient Boosting is an important applicated popular algorithm developed by Tianqi in 2014. And because of its excellent performance on regression and classification problems, it is recommended as the first choice in many cases, such as industry and the Internet applications, which is even implemented in machine learning platforms. However, there are still many challenges in applying it for load forecasting. First, the Extreme Gradient Boosting algorithm relies on many hyperparameters to tune during the model building, and the reasonable hyperparameters directly determine the final prediction effect of the model. For the reason that an optimisation algorithm that can balance both data characteristics and model characteristics is very vital^11,12^. Secondly, when transforming time series into a general supervised regression problem in machine learning, it is complex to construct the data to have both historical memories and ensure the model has sufficient generalization ability after training^13,14^. The Two issues above are the key to combining Extreme Gradient Boosting even for all Machine learning algorithms with load forecasting tasks or time series.

In this study, (1) we completed data exploration for regional power grid consumption demand load data in the Ile-de-France region of France, ensured the best width of the sliding window for the machine learning model by the Discrete Fourier Transform and Pearson Correlation Coefficient methods, and added 5 date features in the dataset for feature engineering work. (2) We designed the TPE-XGBoost algorithm by combining the Tree-structured Parzen Estimator method and the Extreme Gradient Boosting model. By comparing with the original unoptimised model and other 8 machine learning algorithms, our proposed model can effectively improve the prediction performance for power demand load forecasting in the individual testing dataset. (3) We conducted a model evaluation on the TPE-XGBoost model we designed and discussed in detail the feature engineering of the dataset and the modelling effect of the TPE optimisation for the XGBoost model.

## Material and methods

### Loading forecasting dataset and data exploring

Île-de-France (literally "Isle of France") is one of the 13 administrative regions in mainland France and the capital circle of Paris. The average temperature is 11 °C, and the average precipitation is 600 mm. Île-de-France is the most densely populated region of France. According to the 2019 report, this region provides France with a quarter of jobs in total employment, of which the tertiary sector accounts for near nine-tenths of jobs. Agriculture, forests and natural areas cover nearly 80% of the surface. As well, the region, as the first industrial zone in France, includes electronics and ICT, aviation, biotechnology, finance, mobility, automotive, pharmaceuticals and aerospace.

**Figure 1:**
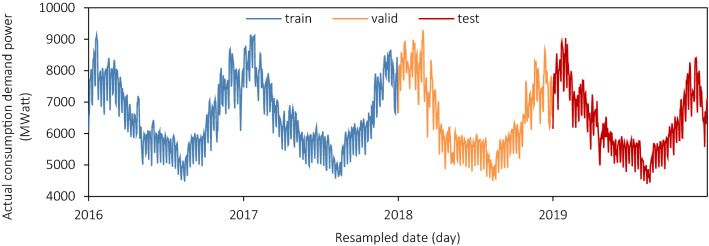
Demand consumption of electrical power (daily, MWatt).

We analysed the power load in the Île-de-France region with a 30-min sampling rate with a total of 70,128 records over four years, from 2016 to 2019. The data is from the éCO2mix API provided by the RTE (Réseau de Transport d'Électricité)^15^. The original data were resampled as the maximum daily power into 1461 records in Fig. 1, whose y-axis is the real-time demand load power (unit: MWatt). As shown below, the trend between the years of the series is similar and has an evident periodicity; each cycle is V-shaped with visible seasonality. Due to the characteristics of power load and the region's actual situation, each cycle's trend is stable without an apparent growth or decay.

The dataset we collected from éCO2mix is divided into three parts, the blue training dataset (2016, 2017), the orange validation dataset (2018) and the red testing dataset (2019) in Fig. 1. The training one builds the main models, and the validation one is analysed for optimisation eval. And testing one will check the models’ performance on several different metrics. The data exploring part of the time series will be finished in the validation one in 2018 to avoid data leakage.

In the following Table 1, we use feature engineering to transform time series into a supervised learning dataset for machine learning as the additional date feature^16^.

**Table 1:** The date features added in the dataset.

Feature index	Data type	Description
Date_feature_1	Integer	Day of the week (Monday = 0 and Sunday = 6)
Date_feature_2	Integer	Day of the month (from 1 to 28 ~ 31)
Date_feature_3	Integer	The day of the year (from 1 to 365/366)
Date_feature_4	Integer	The week of the year (from 1 to 53/54)
Date_feature_5	Integer	The month this year falls on (from 1 to 12)

Finally, loading forecast values at the first N moments will be added to the dataset as a memory feature in the form of a sliding window called momery_feature_1 ~ momery_feature_N. However, the choice of N, the memory length or the time lag is not casual. We will use a method combining Discrete Fourier Transform and Pearson Correlation Coefficient to complete the memory length determination.

### The best width of windows analysis

Data-driven loads forecasting issues of machine learning require the datasets to be produced in the form of sliding windows. Then, the time series issue transforms into a supervised regression in machine learning. And there is a complex effect on the window width or called lags count of the dataset. The longer width of the window, the more abundant the memory information as more features in the sample. However, for the machine learning algorithms based on statistics experience, more features would cause unideal results for practical application by too many irrelevant features. On the other hand, too short a window means fewer features, which might be underfitting for insufficient information.

**Figure 2:**
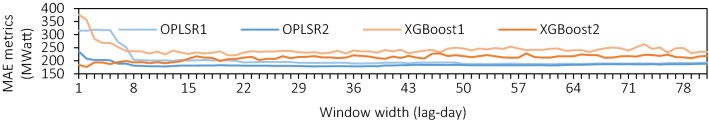
MAE metrics with wider windows of testing dataset.

Figure 2 above is the effect of different window widths in the testing dataset of the XGBoost model and Linear Regression (OPLS), whose x-axis is the window’s width of data features and the y-axis is the mean absolute error (units: MWatt) in the testing dataset (2019), and the models indexed 1 mean no date features adding. It can be seen that the relationship between the performance and window width is not a simple linear relationship. This figure shows a dramatic decline in MAE with wider windows, it reached a low point, and then the MAE fluctuates within a specific range and worsens when the windows widen.

The Fourier Transform is a practical tool for extracting frequency or periodic components in signal analysis. Generally, the synthetic signal $f\left(t\right)$ can be converted to frequency domain component signals $g(freq)$ as below if it satisfies the Dirichlet conditions in the range of $(-\infty ,+\infty )$:

$$ g\left( {freq} \right) = \mathop \smallint \limits_{ - \infty }^{ + \infty } f\left( t \right) \cdot e^{ - 2\pi i \cdot freq \cdot t} d_{t} $$

the power loads time series in this paper are sampled discretely with limited length, and the Fast Discrete Fourier method proposed by Bluestein^17^ is used instead as below:

$$ g\left( {freq} \right) = \mathop \sum \limits_{t = 1}^{N} \left[ {f\left( t \right) \cdot e^{{ - 2\pi i \cdot freq \cdot \frac{t}{N}}} } \right] $$

where $N=365$ is from the validation dataset in 2018, and the $freq$ series contain the frequency bin centers in cycles per unit of the sample spacing with zero at the start. The second half of $freq$ series is the conjugate of the first half, only the positive is saved. And bring $period=\frac{1}{freq}$ back as below:

$$ g\left( {period} \right) = \mathop \sum \limits_{t = 1}^{N} \left[ {f\left( t \right) \cdot e^{{ - 2\pi i \cdot \frac{t}{period \cdot N}}} } \right] $$

**Figure 3:**
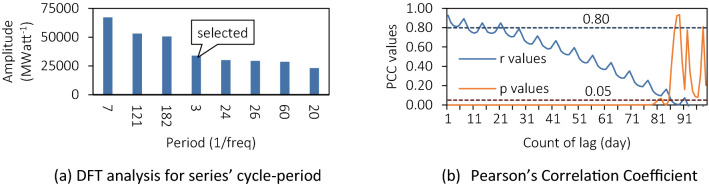
Features engineering of windows width analysis.

Remove the $inf$ and the $period=N$ item $g(period)$, and the first eight amplitude of $period-g\left(period\right)$ bar plots as shown in Fig. 3a^18^. Some of the periods are related to the natural time cycle: 121 as a quarter of a year, 182 as a semi-annual. And not all of the meaning is clear, such as 3, 24 and 26, which are difficult to have sufficient explanation.

Further, the Pearson correlation coefficient (PCCs) are used to calculate a more detailed period. The PCCs measure the linear relationship between two datasets as below:

$$ PCC\left( {{\varvec{x}}\left( {t_{0} } \right),\delta } \right) = \frac{{\sum \left( {{\varvec{x}}\left( {t_{0} - \delta } \right) - \overline{{{\varvec{x}}\left( {t_{0} - \delta } \right)}} } \right)\left( {{\varvec{x}}\left( {t_{0} } \right) - \overline{{{\varvec{x}}\left( {t_{0} } \right)}} } \right)}}{{\sqrt {\sum \left( {{\varvec{x}}\left( {t_{0} } \right) - \overline{{{\varvec{x}}\left( {t_{0} } \right)}} } \right)^{2} \cdot \sum \left( {{\varvec{x}}\left( {t_{0} } \right) - \overline{{{\varvec{x}}\left( {t_{0} } \right)}} } \right)^{2} } }} $$

where the ${\varvec{x}}({t}_{0})$ is the series to predict as y target of dataset and the ${\varvec{x}}\left({t}_{0}-\delta \right)$ is the $\delta$ lag series of ${t}_{0}$. The larger PCC means there are more correlated relationships between two series.

In Fig. 3b, the X-axis is the time interval numbers and the Y-axis is the Pearson’s Correlation Coefficient values (blue) and Two-tailed p-value (orange). According to experience in the general statistical sense^19^, when the Pearson Coefficient is greater than 0.80 (blue dotted line), it can be considered that the two series have a strong correlation; when the p-value is less than 0.05 (orange dotted line), the hypothesis is established. The orange curve shows that the about first 50 memory features have a positive correlation with the predicted target, so the minimum period should be less than 50. Furthermore, Memory-feature 1 ~ 5 have Pearson’s correlation coefficient values greater than 0.80; that is, the values within 5 are strongly correlated. And the number of periods in the FFT to satisfy this value requirement is 3, therefore, our model will use 3 as the window width.

We will further compare the three kinds of widths, 7, 14 and 28, as a control to complete the sequential modelling.

### Extreme gradient boosting optimised by tree-structured Parzen estimator

Gradient Boosting originates from the paper by Friedman in 2011^20^. XGBoost is an open-source software library of extreme gradient boosting developed by CHEN Tianqi^21^ that ensembles tree models by a series of strategies and algorithms such as a greedy search strategy based on gradient boosting. As an additive ensemble model, XGBoost considers the gradient of first-order derivative and second-order derivative in the Taylor series for the loss function and constructs in the case of probability approximately correct (PAC). The objective function is as follows:

$$ obj\left( t \right) = \mathop \sum \limits_{i = 1}^{n} loss\left( {y_{i} ,\hat{y}_{i}^{t - 1} + f_{t} \left( {x_{i} } \right)} \right) + \mathop \sum \limits_{i = 1}^{t} {\Omega }\left( {f_{i} } \right) $$

where the $t$ means the rounds of ensemble processing and the $\omega$ means the regularisation part.

Take a second-order Taylor expansion on the loss function and add the parameters of the tree structure in the regular term, Then the objective function transforms into below:

$$ obj\left( t \right) = \mathop \sum \limits_{i = 1}^{n} \left[ {g_{i}^{t} \cdot f_{t} \left( {x_{i} } \right) + \frac{1}{2} \cdot h_{i}^{t} \cdot f_{t} \left( {x_{i} } \right)^{2} } \right] + \gamma \cdot T + \frac{1}{2} \cdot \lambda \cdot \mathop \sum \limits_{j = 1}^{T} \omega_{j}^{2} $$

where the $g$ and the $h$ is the derivative term of the loss function; the $T$ and $\omega$ are the parameters of ensembled decision trees’ structure parameters; $\gamma$ is the minimum loss required for further partitioning on the leaf nodes of single tree; $\lambda$ is the L2 regularization term.

A greedy strategy to solves the $obj(t)$ for a local optimal solution $\upomega =-\frac{G}{\lambda +\mathrm{H}}$ then Bring back:

$$ best_{{obj\left( t \right)}}  =  - \frac{1}{2} \cdot \sum\limits_{{j = 1}}^{T} {\left( {\frac{{G_{j}^{2} }}{{H_{j}  + \lambda }}} \right)}  + \gamma  \cdot T $$

With the meta, weak learner $t$ generated in each round, $bes{t}_{obj\left(t\right)}$ is used as the basement strategy for the growth of the decision tree, which controls the generalisation ability for the boosting process.

Most specific detail for XGBoost can refer to the paper, *XGBoost: A scalable tree boosting system*^21^.

**Figure 4:**
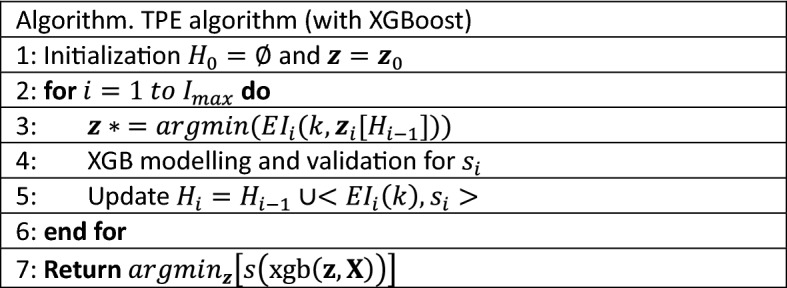
Pseudocode of Tree-structured Parzen Estimator optimising XGBoost.

XGBoost is a powerful ensemble algorithm, and there are numerous hyper-parameters to tune for the best performance in application, however, it is a black-box process widely recognised^22^. We adopt the Tree-structured Parzen Estimator^23^ (TPE), one of the sequential model-based optimisation methods (SMBO) based on the Bayesian theorem, to optimise our XGBoost model for time-series forecasting. The TPE pseudocode as below shown in Fig. 4:Where the ${\varvec{z}}$ is the set of hyperparameters of the search space, the $s$ is the metrics score of XGBoost with ${\varvec{z}}$ in the validation dataset, and the $H$ is the history of validation scores and the selected ${\varvec{z}}$.

The EI is the core of TPE, which builds a probability model of the objective function and uses it to select the most promising hyperparameters to evaluate in the true objective function^23^:

$$ EI = \frac{{\mathop \smallint \nolimits_{ - \infty }^{ + \infty } \max \left( {{\varvec{s}}{*} - {\varvec{s}}\left[ {H_{i - 1} } \right],0} \right)p\left( {{\varvec{s}}\left[ {H_{i - 1} } \right]} \right)ds{ }}}{{k + \frac{{\left( {1 - k} \right)g\left( {{\varvec{z}}\left[ {H_{i - 1} } \right]} \right)}}{{l\left( {{\varvec{z}}\left[ {H_{i - 1} } \right]} \right)}}}} $$

the $l({\varvec{z}})$ is the value of the objective function less than the threshold $k$, and $g({\varvec{z}})$ is the objective function greater than the threshold.

We first build the XGBoost model (XGBoost) by default values in Table 2, and then nine hyper-parameters in Table 2 are going to be optimized in the searching space below by the TPE algorithm for TPE-XGBoost models.

**Table 2:** Hyperparameters to be optimised of XGBoost in paper.

Hyperparameter to tuning	Data type	Default value	Searching space
Alpha	Float	0	0.01–1.00
Learning rate	Float	0.3	0.01–0.20
Max depth of single tree	Integer	6	2–5
Minimized child weight	Float	1	0.50 to 0.60
Minimized split loss	Float	0	1e-10–1.00
Subsample	Float	1	0.90–1.00
Col sample by tree/level/node	Float	1	0.90–1.00

Alpha: a regularization parameter in meta learners’ ensemble; Decreasing it will give model looser constraints. And this is the only hyperparameter in this paper to control model in the ensemble level by regularization.

Learning rate: a weight parameter for new meta learners to slow down the boosting ensembles; smaller learning rate would make ensemble slower but more conservative.

Max depth of single tree: a parameter of meta models struction; deeper tree would more complex but more likely overfitted.

Minimized child weight: a parameter of meta models struction by controlling leaf nodes. If a leaf node with the sum of instance weight less than it, the node will be given up as a leaf; Too small weight would make ensembled model easy to underfit.

Minimized split loss: a parameter for meta models building by leaf nodes construction. The nodes would be abandoned if the loss less than this parameter; The larger it is, the more conservative the algorithm would be.

Subsample: it is samples counts ratio of subset for training; A balanced Subsample can prevent overfitting, but too small subsample would make models hard to satisfy application.

Col sample by tree/level/node: it is a subsample for features as Subsample above did.

**Figure 5:**
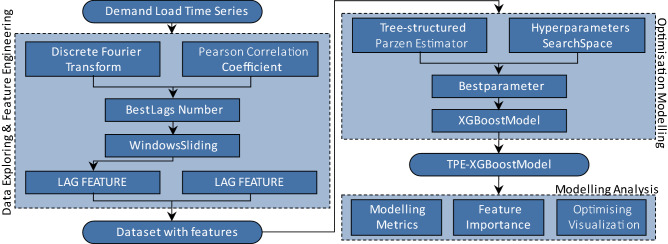
Feature Engineering and Optimised Modelling Process.

We limit the tuning iterations to 500 and the target of each iteration will be set of the MAPE in the validation dataset (2018). And the Fig. 5 is the whole flow process in this paper.

## Results

### Prediction models of testing dataset

XGBoost and TPE-XGBoost we recommended have been modelled in four kinds of windows width data: 3d, 7d, 14d and 28d in train dataset (2016, 2017). In addition to this, eight other below machine learning algorithms have been conducted as comparative experiments on at the same time, including:

- Three ensemble models: Gradient Boosting Decision Tree models (*pGBDT*) and Adaptive Boost models (Adaboost) based on scikit-learn; Random Forest models (*RandomForest*) based on XGBoost.
- One linear model: built by Ordinary Least Squares Method (*OPLSR*).
- One Support Vector Machine model: based on libsvm algorithm with a Radial basis function kernel (*RBF-SVR*).
- One Neural Network model: Perceptron model (*Perceptron*) with triple hidden layers shaped (256, 128, 64) built by scikit-learn framework.
- One Neighbours Model: K-Nearest Neighbours model (*KNN*) with Euclidean distance metrics.
- Single Decision Tree model: Tree (*SingleTree*) model built by scikit-learn framework without max depth limit as the contrast of ensemble models.

**Figure 6:**
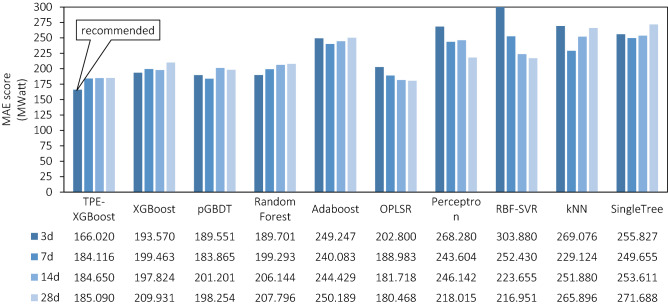
Mean Absolute Error values (MAE values).

Figure 6 shows mean absolute error values (MAE values) respectively, where the top model valued at 166.02 is the XGBoost optimised by the TPE algorithm with data wide of 3d.

Obviously, TPE method does improve XGBoost performance. All MAE metrics of four XGBoost models trained with different windows width data are apparent to improve after being optimised by the TPE algorithm. They decrease from 193.57, 199.46, 197.82, 209.93 to 166.02, 184.11, 184.65, 185.09 respectively, whose optimization achieves 14.23, 7.70, 6.66, and 11.83%.

The MAEs of Five ensemble learning methods (TPE-XGBoost, XGBoost, pGBDT, Random Forest and Adaboost) get a slight rise with longer windows. This proves from the side that proper selection of window width is vital for such ensemble learning models to predict time series correctly. However, OPLSR, Perceptron, and RBF-SVR models have the opposite trend after training with more previous features.

But as Fig. 2 shown, this trend would reach a limit value not as good as TPE-XGBoost models’ scores and then it will begin fluctuating in ranges. The best MAE during the 1–81 is 178.539 (width = 31), which gaps obviously with our TPE-XGBoost with 166.02. We will discuss in-depth for this phenomenon in the next part of our paper.

Another three metrics (MAPE, R2 and MaxError) from the testing set are listed partly in Table 3. Shorter windows of our method can reach higher on two overall metrics: MAPE = 2.61%, R2 = 0.9471. And the max residual metrics don't result in ideal results ranged 1183–1436. However, the 3d-TPE-XGBoost’s max residual is still in an acceptable value on par with the best 14d-OPLSR model, scored 895, and the TPE process suppresses it compared to XGBoost not optimised to a certain extent in 7d, 14d and 21d data.

**Table 3:** Results metrics from testing dataset of models.

		TPE	XGBoost	Random	OPLSR	Perceptron	RBF-SVR
		XGBoost		Forest			
MAPE	3d	**2.61%**	3.04%	2.99%	3.19%	4.19%	4.80%
	7d	2.88%	3.09%	3.16%	2.98%	3.82%	3.94%
	14d	2.90%	3.06%	3.26%	2.84%	3.77%	3.49%
	28d	2.90%	3.29%	3.28%	2.80%	3.36%	3.38%
R2 score	3d	**0.9471**	0.9328	0.9355	0.9307	0.8885	0.8430
	7d	0.9389	0.9297	0.9317	0.9388	0.9052	0.8978
	14d	0.9413	0.9319	0.9271	0.9419	0.8983	0.9153
	28d	0.9387	0.9255	0.9261	0.9421	0.9198	0.9208
Maximum residual	3d	**1183**	1174	1294	975	1506	1448
	7d	1436	1550	1305	979	1190	1125
	14d	1209	1249	1226	895	1450	1216
	28d	1257	1529	1230	919	1126	1082

Significant values are in [bold].

**Figure 7:**
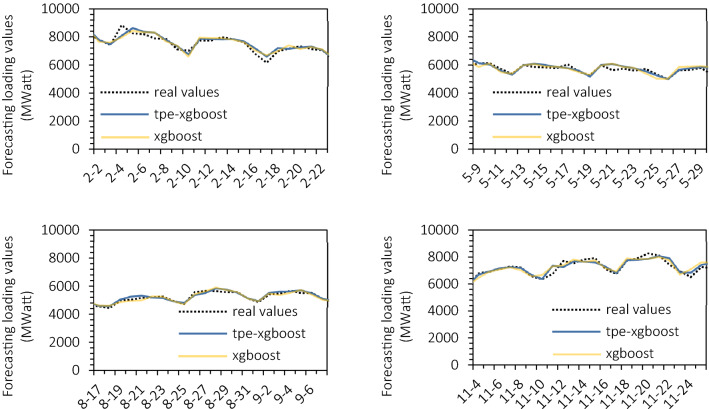
Forecasting and true values of testing dataset in 2019.

Figure 7 is the sequence comparison figure of random selected 21-day predicted and real values among four seasons of 2019. The model of 3d we proposed can make excellent predictions of the periodic and frequency trend of the real time series, mutually confirmed by its better MAE, MAPE, and R2 metrics.

The TPE-XGBoost model performs well before December. A suppressing fixing by TPE can be observed compared to the unoptimised XGBoost method, especially in January, April and June. The December series forecast is terrible. This month, the negative impact of almost all models is contributed by the max residual of 3d-TPE-XGBoost.

### TPE optimisation processing for XGBoost models

Three tuning methods that were also applied to the Boosting method widely are chosen as the control group. All four methods have the same search space (Table 2), and the input feature of dataset are 3d, which is as the style with the results of above section. Three control group with XGBoost is as follows:

- Random Search^24^: Random search, also called black-box methods, is a family of numerical optimization methods that do not require the gradient of the problem to be optimized, and RS can hence be used on functions that are not continuous or differentiable.
- Simulated annealing^25^: Simulated annealing is a probabilistic technique for approximating the global optimum of a given function. Specifically, it is a metaheuristic to approximate global optimization in a large search space for an optimization problem.
- Evolution Strategy: We chose Lightweight Covariance Matrix Adaptation Evolution Strategy^26^, where CMA-ES is a stochastic, or randomized, method for real-parameter (continuous domain) optimization of non-linear, non-convex functions.

Table 4 is the results of the optimisation processing. And the TPE row is the same as above sections. Compared with vanilla XGBoost + 3d (MAE = 194), the model optimized by tuning methods has better performance generally from metrics. This proves that tuning hyperparameters for model optimization has the effect of improving model performance without the changing of core Boosting algorithms generally.

**Table 4:** Results of optimisation processing.

	MAE	MAPE	R2	MaxError
TPE	**166**	**2.61%**	**0.9471**	**1183**
Random search	169	2.65%	0.9465	1134
Simulated annealing	172	2.70%	0.9453	1246
Evolution strategy	171	2.68%	0.9456	1227

Significant values are in [bold].

The TPE method we recommended have achieved the best results in three of the four metrics and the second best in the MaxError, which proves the effectiveness of the method. Indirectly, it can also be preliminarily demonstrated that the SMBO method has better applicability for regional loading forecast.

**Figure 8:**
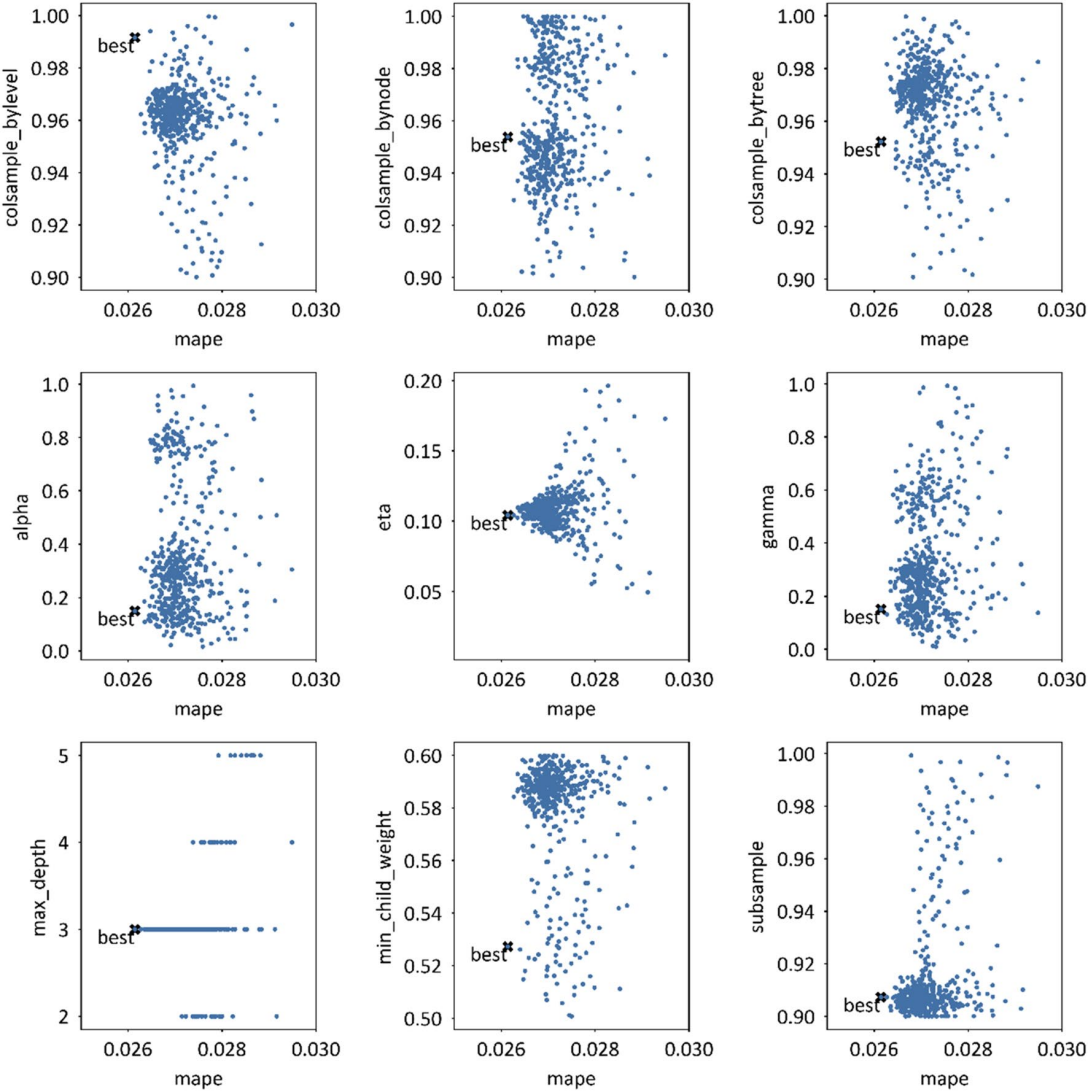
Nine Tuning hyperparameters with MAPE values.

The visualization process of nine hyperparameter tuned by the TPE algorithm is shown in Fig. 8. The object value for loop iterations is set to the MAPE (mean absolute percentage error) from the validation dataset in 2018, which is neither included in the training nor the testing dataset. After 500 iterations of learning, the model can gain an acceptable excellent target in the validation set of MAPE = 0.02647. With the iterations increasing, the validation target is gradually distributed to a tight range. The max depth of the trees in XGBoost is selected to 3 in a range from 2 to 5; the learning rate(eta) is around 0.11 from 0.02 to 0.2.

The best hyperparameter set appearing in the 499th iteration of 500 rounds is listed in Table 5. More searching rounds would gain a better MAPE of validation set but it needs more time to run.

**Table 5:** Best hyperparameters of XGBoost optimised by TPE in paper.

Eta	Alpha	Gamma	Subsample	Max depth
0.10411162943929	0.14962743583968	0.1513032788308	0.90727951205518	3
Min child weight	Colsample by level	Colsample by node	Colsample by tree	Iteration
0.52728463623425	0.99153864007020	0.95380256761024	0.95222870269938	499/500

**Figure 9:**
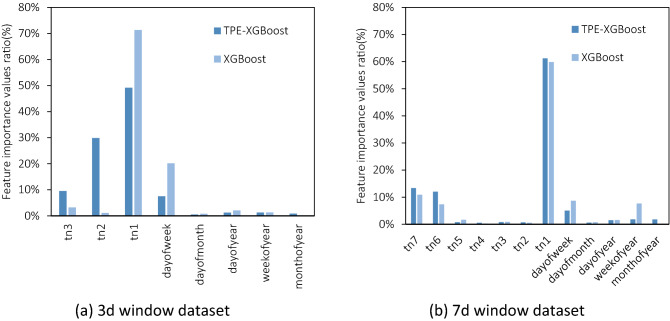
Feature importance percentages of XGBoost models.

As an ensemble algorithm based on the tree model, FI (feature importance rate) stands for the weights in the modelling of features in the dataset. Figure 9 shows the two different width series (3d we preferred and longer 7d) of TPE-XGBoost and the Unoptimized one. The models optimized have higher FI values of the features before tn1 time of 3d one: almost 40% FI values of tn2&tn3 but the unoptimized one’s less than 10%. Wider windows models focus more on tn6 and tn7, and the TPE processing rises the rate of FI values of them and suppresses the contribution of the tn2 ~ tn5.

## Discussion

- The power load data has a clear time continuity. That is the load data will not change abruptly only in the case of extreme events (such as grid crashing, etc.). This is the reason why linear regression (OPLSR) and the simplest model perform still well for the wider windows. The XGBoost method, even most machine learning methods, is based on historical data and does not have the concept of temporal continuity. We make sliding windows to provide the memory for them and so transformed time series problems into regression problems, trains and forecasts data will reference through this window feature. As for the TPE method, from the discussion of FI above, it can be seen that it suppresses the modelling weight of the near memory features, and increases the model's attention to farther ones. It is the immediate reason why the TPE method can improve modelling performance by hyperparameters controlling.
  We believe that it is necessary to use the minimum period as the window width, which is a targeted treatment of the continuity characteristics of time series or load forecasting data. The window with the shortest period includes at least the complete memory of the data of interest and does not contain redundant information of multiple periods. Although a wider window will provide richer historical information, the XGBoost algorithm's focus on data continuity will suffer, which is regulated to control the risk of generalization.
  We believe that the main impact of adding the date features to the model is to ensure that the algorithm can have the ability to extract other periodicities. Time series data, including our load data, is of course highly cyclical, and the cycles it contains may be related to the real world with clear explanations. Monthly and weekly data are also cyclical, with stronger or weaker correlations between these cycles. Therefore, we have added five date features to the feature engineering. The five date features can help the algorithm to extract information from multiple cycles as much as possible. If there is no data feature, machine learning methods will maybe not perform effective fitting and prediction on periodic time series.
- TPE-XGBoost as mentioned above is a two-step process with modelling-then-validation, that is, given the hyperparameter search space of XGBoost, adjusted and optimized by TPE for finally taking the increase or decrease of a certain target objective value as the goal. In the optimization process, which kind of metrics to use and where the metrics are from are two crucial issues.
  Usually, the optimization target metrics are one of the metrics for evaluating the modelling. Such as MAE or RMSE, two reasonable targets can both describe the error value between predicted data and real data from a certain scale. However, the metrics for optimization are different from evaluating purposes. In our TPE-XGBoost algorithms, setting MAPE as the target value through the data modelling from 2016 to 2017 and predicted of true values of 2018 by 500 iterations, the MAPE can effectively improve MAE\RMSE\R2, etc. Other evaluation metrics can only improve their own performance in an independent test dataset. Other metrics are not unchanged but are less obvious than MAPE.
  In addition, the usual source of objective values is the k-folds Cross-validation of the training set itself, this way can maximize the use of existing sequences for modelling and evaluation when the data is not sufficient. However, this method, as literally stated, needs to use 5 times the calculation of single modelling for repeated fitting, and the obtained k-folds have great differences in the dataset in this paper no matter what metrics are used, resulting in a slow and ineffective optimization process. In fact, for the load forecasting request itself, the data is abundant, and even several years of historical data can be traced back at power grid operators, and there is no problem of insufficient data. In the machine learning method, if such a dataset is used for the validation of the model, it is necessary to ensure that the training data and the validation data should be independent and identically distributed, and our training dataset, validation dataset and test dataset, no doubt, are all in the form of there is actually real-world data, and the data itself is consistent, so our approach of using an independent validation set is correct.
  Therefore, as described in this paper's results and discussion, we propose to adopt MAPE metrics from a separate validation dataset as objective values in the optimization process of TPE-XGBoost.
- This paper does not consider the introduction of external variables, and only studies from the time series itself, but it also achieves reliable forecasting results. This is because the external variables such as temperature, wind speed and social practice commonly used in load forecasting problems can be replaced by the date feature we introduced. These external variables are also periodic and to be predicted, and the function of the date feature is to provide a calibration reference for the memory of the time series from another aspect. Such a calibration reference that has an independent and identical distribution in the dataset is more valuable than the actual wind speed and other data.

## Conclusions

The results in this paper show that (1) fewer window features are capable to revert the power loads time series we are concerned about. The three-day width of windows analysed from FFT and Pearson correlation have enough information to do better than longer ones. (2) XGBoost as a practical and effective algorithm can achieve the forecasting task with fewer features, however, there is remarkable necessary to add optimising processing by the TPE method. Hyperparameters from TPE will get the most out of the performance of XGBoost with short windows.

In summary, the optimal window width of the time series data is determined in this study through Discrete Fourier Transform and Pearson Correlation Coefficient Methods, and five additional date features are introduced to complete feature engineering. And TPE optimisation ensures nine hyperparameters’ values of XGBoost and improves the models obviously. By fitting demand series from 2016 to 2018, the TPE-XGBoost model we designed has an excellent performance of MAE = 166.020 and MAPE = 2.61% in 2019 of single lagging. Compared with the original model, the two metrics are respectively improved by 14.23 and 14.14%; compared with the other eight machine learning algorithms, the model performs with the best metrics as well.

## Data Availability

Data are available from the éCO2mix of Français Réseau de Transport d’Electricité(French language) website at https://www.rte-france.com/eco2mix , or get the copy at https://github.com/gniqeh/TPE-XGB-TS by MIT License.
